# Annual Commencement Exercises in the Buffalo Medical College

**Published:** 1868-02

**Authors:** 


### Editorial Department.

### Annual Commencement Exercises in the Buffalo Medical College. List of Graduates.

The annual commencement exercises of the Buffalo Medical College, took place February 25th, at the Opera House, in the presence of a large audience. Prof. J. P. White presiding.

The following Curators were present:—Drs. Hovey and Montgomery of Rochester, Lauderdale of Geneseo, Strong of Westfield, Baker of Warsaw, Flood of Elmira, Townsend of Bergen, Craig of Churchville, Menzie of Caledonia, Bennett of Erie, Pa., Eddy and Samo of Buffalo, and Lapham of Aurora.

At the meeting of the Council of the University, Dr. Geo. E. Hayes, of Buffalo, was elected Member to fill vacancy caused by death of the late Jesse Ketchum, Esq.

*Curators.*—At the same meeting the following gentlemen were elected Curators: Dr. C. C. Wyckoff, of Buffalo; Dr. Harvey Montgomery, of Rochester; Dr. Francis Burdick, of Johnstown, Fulton county; Dr Robert Menzie, of Caledonia; Dr. A. W. Fuller, of Le Roy.

Hon. Millard Fillmore, Chancellor of the University, conferred the degree of *Doctor of Medicine* upon the following gentlemen:

John Morton McWharf, Java Village, Wyoming county, N. Y.
Miles Gaylord Myers, Breesport, Chemung county, N. Y.
Frederick W. Hogarth, Smithport, McKean county, Pa.
Hiram Parker Trull, Varysburg, Wyoming county, N. Y.
John Krebiel, New Haven, Huron county. Ohio.
William James Barry, St. Catherines. Ontario.
Frank E. Bliss, Eagle, Wyoming county, N. Y.
Malcom Ney McNaughton, Galesburg, Kalamazoo county, Mich.
John Mulvany, Dunnville, Ontario.
George Barry Jones, Corfu, Genesee county, N. Y.
Dwight C. Chase, Lima, Livingston county, N. Y.
Eber Smith Carlisle, jr., Johnson, Jones county, Iowa.
Arthur Martin Gerow, Sterling, Hastings county, Ontario.
Luke Arthur Harcourt, Arthur, Wellington county, Ontario.
Henry Lane McCoy, Smithport. McKean county, Pa.
Emmet Fleming Pyle, Pekin, Niagara county, N. Y.
Albert Harlow Flood, Elmira, Chemung county, N. Y.
Wm. Urias Truckenmiller, McEwensville, Northumberland county, Pa.
John Fletcher Falling, Lyons, Wayne county, N. Y.
Oscar H. Hall, Marilla, Erie county, N. Y.
Loran Fernando Boies, Aurora, Erie county, N. Y.
Luke Chandler Higgins, Corfu, Genesee connty, N. Y.
Cassius Marcellus Ackley, South Dansville, Steuben county, N. Y.
Frank Granger Osborn, Holland, Erie county, N. Y.
David Estep Chace, A. B., Buffalo, Erie county, N. Y.

Oran Amasa Dean, Rochester, Monroe county, N. Y.
Joseph Quinlan, Buffalo, Erie county, N. Y.
Proteus Posket Bielby, Little Falls, Herkimer county, N. Y
Matthew Willoughby, Buffalo, Erie county, N. Y.
Frank W. Crane, Corfu, Genesee county, N. Y.
Henry S. Ellwood, Buffalo, Erie county, N. Y.
Galette B. Gilbert, Alexander, Genesee county, N. Y.
Henry Platner Hall, Sinclairville, Chautauqua county, N. Y.
George Washington Allen, Urbana, Champaign county, Ill.
Claude Wheeler, Farmer Village, Seneca county, N. Y.
Elliott H. Woolsey, Perrington, Monroe county, N. Y.
Francis Martin, Mount Read, Monroe county, N. Y.
Robert Fowler Dunlap, Darlington, Beaver county, Pa.
Patrick Francis Madden, Buffalo, Erie county, N. Y.
John Dumbach, Louisiana.

The honorary degree of Doctor of Medicine was conferred upon Theodore Evans, of Paris France.

The charge to the graduating class was delivered by Dr. J. F. Miner. Dr. Matthew Willoughby, member of the graduating class, gave the Valedictory Address, which was well written and very appropriate for the occasion.

The graduates, in the general examination, acquitted thomselves with high honors, and as a general rule, gave promise of future honor and usefulness. The rigid and careful sifting of merit by both the Faculty and Curators proved them ever faithful janitors, guarding with jealous care the entrance to the medical profession.

*To Prof. J. F. Miner:*

At a meeting of the members of the graduating class, held immedialely after the close of the commencement exescises, upon motion, a resolution was adopted, that Prof. Julius F. Miner be requested to allow the publication of his "Address to the Members of the Graduating Class" in the Buffalo *Medical and Surgical Journal.*

PROTEUS P. BIELBY,

J. W. WILLOUGHBY,

DAVID E. CHACE,

*Committee.*

BUFFALO, February 25, 1868.

---

*To Drs. Bielby, Willoughby and Chace:*

GENTLEMEN: I have to thank you and those you represent, for your very polite invitation to allow the publication of my address before the graduating class. I had proposed to withhold it from publication, but a strong desire to gratify the wishes of yourselves and associates, and also for the purpose of correcting the fearful errors of "short-hand reporters," who have represented me as saying precisely the reverse of what I did say, or would be willing to say, may so far change my purposes in this respect as to allow its publication, according to the request of the class.

With much regard for yourselves and associates,

I am ever, yours truly,

J. F. MINER.

